# Novel Insights on Lipid Metabolism Alterations in Drug Resistance in Cancer

**DOI:** 10.3389/fcell.2022.875318

**Published:** 2022-05-13

**Authors:** Ruixue Yang, Mei Yi, Bo Xiang

**Affiliations:** ^1^ Hunan Cancer Hospital and the Affiliated Cancer Hospital of Xiangya School of Medicine, Central South University, Changsha, China; ^2^ Hypertension Center, FuWai Hospital, State Key Laboratory of Cardiovascular Disease, National Center for Cardiovascular Diseases, Peking Union Medical College, Chinese Academy of Medical Sciences, Beijing, China; ^3^ Department of Dermatology, National Clinical Research Center for Geriatric Disorders, Xiangya Hospital, Central South University, Changsha, China; ^4^ The Key Laboratory of Carcinogenesis of the Chinese Ministry of Health, Cancer Research Institute and School of Basic Medical Sciences, Central South University, Changsha, China; ^5^ The Key Laboratory of Carcinogenesis and Cancer Invasion of the Chinese Ministry of Education, Cancer Research Institute and School of Basic Medical Sciences, Central South University, Changsha, China

**Keywords:** drug resistance, Cancer chemoresistance, fatty acid metabolism, lipid metabolism, multi-drug resistance

## Abstract

Chemotherapy is one of the primary treatments for most human cancers. Despite great progress in cancer therapeutics, chemotherapy continues to be important for improving the survival of cancer patients, especially for those who has unresectable metastatic tumors or fail to respond to immunotherapy. However, intrinsic or acquired chemoresistance results in tumor recurrence, which remains a major obstacle in anti-cancer treatment. The high prevalence of chemoresistant cancer makes it urgent to deepen our understanding on chemoresistance mechanisms and to develop novel therapeutic strategies. Multiple mechanisms, including drug efflux, enhanced DNA damage reparability, increased detoxifying enzymes levels, presence of cancer stem cells (CSCs), epithelial mesenchymal transition (EMT), autophagy, ferroptosis and resistance to apoptosis, underlie the development of chemoresistance. Recently, accumulating evidence suggests that lipid metabolism alteration is closely related to drug resistance in tumor. Targeting lipid metabolism in combination with traditional chemotherapeutic drugs is a promising strategy to overcome drug resistance. Therefore, this review compiles the current knowledge about aberrant lipid metabolism in chemoresistant cancer, mainly focusing on aberrant fatty acid metabolism, and presents novel therapeutic strategies targeting altered lipid metabolism to overcome chemoresistance in cancer.

## 1 Introduction

**FIGURE 1:**
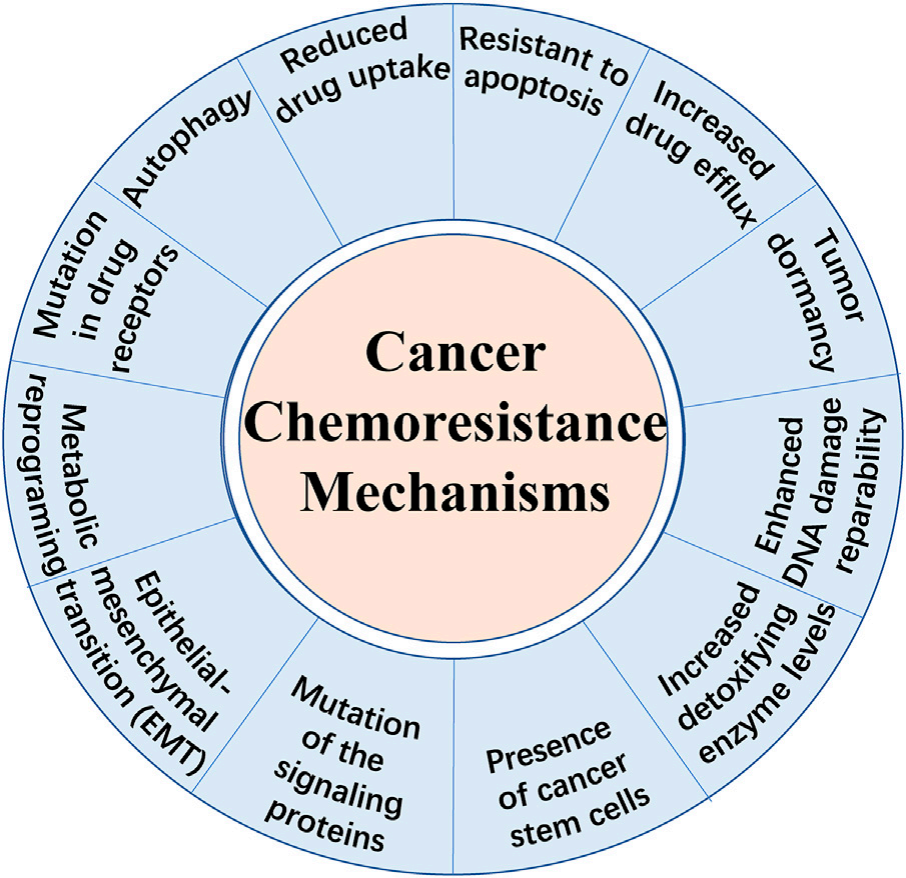
Schematic representation of the main mechanisms of chemoresistance in cancer cells.

Cancer is fundamentally a disorder of uncontrolled cell growth, continual unregulated proliferation, blockade of differentiation and resistant to cell death caused by genetic and epigenetic abnormalities (Greaves and Maley, 2012). Despite great progress in diagnostic methods and therapeutic strategies, therapy failure leads to the death of a large number of patients. Besides immunotherapy which is another new approach that stimulates immune cells to enhance their anticancer activity, chemotherapy remains to be one of the primary methods for cancer treatment. There are different kinds of chemotherapeutic agents including alkylators, antibiotics, antimetabolites, topoisomerases inhibitors, mitosis inhibitors and others (Espinosa et al., 2003; Pan et al., 2016). However, apart from the cell toxicity for normal cells, the effect of chemotherapeutic treatments is limited due to chemoresistance, known as multidrug resistance (MDR). It is broadly recognized that drug resistance is the main cause of tumor recurrence and a major obstacle against tumor control. Overall, there exist two types of chemoresistance in cancer: one is intrinsic drug resistance which is mainly due to high cellular heterogeneity within the tumor bulk and the other is acquired drug resistance which is often induced by therapy (Pan et al., 2016). Until recently, several mechanisms of drug resistance have been highlighted (shown in Figure 1), including increased drug efflux, altered cell cycle, enhanced DNA damage repairmen, insensitivity to death stimuli and tumor dormancy (Szakacs et al., 2006; Assaraf et al., 2019). Notably, metabolic reprogramming has been emphasized to be a novel characteristic preventing cancer cells from chemotherapy-induced death (Hirpara et al., 2019). Aberrant lipid metabolism observed in chemoresistant malignant cells plays an important role in cancer survival and poor prognosis (Wang T. et al., 2018; Lee M. Y. et al., 2018). However, we are far away from comprehensive understanding the underlying mechanisms. Besides, here exists a strong need to identify novel therapeutic strategies to treat chemoresistant tumors. Hence, this review highlights the current knowledge about the role of aberrant lipid metabolism mainly focusing on fatty acids (FAs) in cancer chemoresistance and provides novel strategies targeting specific enzymes or other pathways.

## 2 Mechanisms Involved in Chemoresistance

### 2.1 Increased Drug Efflux

Enhanced drug efflux is one of the most widely studied mechanisms contributing to drug resistance, which is mainly regulated by the ATP binding cassette (ABC) superfamily to reduce the intracellular drug levels (Eckford and Sharom, 2009). The ABC transporters contain seven sub-families designated A to G (Kathawala et al., 2015). Members of ABC transporters are frequently overexpressed in human cancers, which links to drug resistance and unfavorable clinical outcome (Fletcher et al., 2010). For instance, ABCF2 overexpressed in cisplatin-resistant ovarian cancer cells (Bao et al., 2017) and ABCC5 overexpressed in gemcitabine-resistant pancreatic cancer cells (Hagmann et al., 2010) inducing the poor prognosis of the treatments. Selonsertib (GS-4997), targeting apoptosis signal-regulating kinase 1 (ASK1), reversed chemoresistance by suppressing drug efflux activity of ABCB1 and ABCG2 transporters (Ji et al., 2019). This study showed that selonsertib interacted with substrate-binding sites to inhibit efflux function and increase intracellular accumulation of anticancer drugs thereby sensitizing cancer cells.

### 2.2 DNA Damage Repairment

Alkylating agents such as platinum agents kill cancer cells by inducing DNA damage (Jeggo et al., 2016). DNA damage repair is utilized by cells to overcome the multiple DNA damages occurring endogenously and exogenously, which is also the strategy for cancer to adapt and resist to the chemotherapies. For instance, up-regulation of RAD6, a ubiquitin-conjugating enzyme regarded as a DNA repair protein, facilitated DNA damage repair and induced carboplatin-resistance (Somasagara et al., 2017). Rac1, a GTP binding protein, also triggered DNA damage repair by increased nucleotides metabolism to support neoadjuvant chemotherapy (NAC) resistant breast tumors, while silencing Rac1 expression by nanoparticles resensitized the cancer cells (Li et al., 2020). As a crucial mechanism of cancer chemoresistance, DNA repair capacity is considered as a hallmark to predict the prognosis of cancer patients.

### 2.3 Altered Cell Cycle

The dysregulation of cell cycle is intensively involved in tumorigenesis and cancer chemoresistance. On the one hand, cell cycle arrest often leads to insensitivity of tumor chemotherapy. Though fast-dividing cancer cells give anti-cancer drugs more opportunities to contact and damage the nuclear DNA (Zhao, 2016), cancer cell is likely to stay quiescent and enter G0 phase to avoid being killed by anti-cancer drugs. It is considered to be one of the reasons why cancer stem cells (CSCs) challenge the treatment of chemotherapy. CSCs are relatively quiescent cells to stay in the “safe” states evading the attack of anti-cancer drugs (Zhao, 2016). On the other hand, the dysregulation at cell cycle checkpoints makes cell lose the ability to prevent genomic instability and halt cell cycle progression, leading to cancer chemoresistance consequently (Horibe et al., 2015).

### 2.4 Autophagy

Autophagy is a conserved metabolic recycling mechanism intensively implicated in cancer development (Levy et al., 2017). Autophagy may exert a tumor suppressive role at early stage by eliminating damaged organelles and protein aggregates, safeguarding genome stability. Paradoxically, when a tumor occurs, autophagy has the opposite effects (Levy et al., 2017). Cancer cells often have higher basal level of autophagy when compared to its normal counterparts. Under the stress condition induced by chemotherapy in tumor cells, autophagy usually appears to have effects on promotion of cell survival and resistance of anti-cancer drugs (Huang et al., 2016). Verteporfin, an autophagy inhibitor, sensitizes pancreatic ductal adenocarcinoma to gemcitabine, suggesting that inhibition of autophagy is a promising approach to overcome chemoresistance (Donohue et al., 2013).

### 2.5 Epithelial Mesenchymal Transition

Epithelial mesenchymal transition (EMT) refers to a biological process originally observed during embryogenesis, in which epithelial cells turn into mesenchymal phenotype cell by specific program (Lamouille et al., 2014). EMT presents in cancer cells to promote stemness, migration, invasion and dissemination (Ye and Weinberg, 2015). Whereas, mesenchymal-epithelial transition (MET), the reverse process of EMT, induced the cessation of migration and facilitated metastatic colonization of cancer cells to form the new tumor (Nieto et al., 2016). Transcriptional and translational/post-translational control regulates EMT at multilayer levels mainly including SNAIL, TWIST and ZEB families (Brabletz et al., 2018). Emerging evidence suggests that EMT contributes to chemoresistance in cancers. In pancreatic ductal adenocarcinoma, EMT program was found to protect cancer cells from cytotoxicity of gemcitabine by suppressing proliferation and drug transporters, therefore inducing chemoresistance (Zheng et al., 2015). EMT is also considered to play an important role in the formation of a stem cell phenotype (Alison et al., 2012). In brief, EMT regulates chemoresistance by promotion of CSCs signaling pathways, avoidance of drug-induced apoptosis and microenvironment remodeling (Du and Shim, 2016).

### 2.6 Cancer Stem Cells

Cancer stem cells (CSCs) are sub-populations of cells which possess the ability of self-renewal and differentiation, appearing in most tumors (Alison et al., 2012). CSCs have the ability to undergo asymmetric cell division or symmetrical division to facilitate heterogeneity in cancer (Alison et al., 2012). Importantly, CSCs, considered as the “root of cancer” to promote cancer progression and metastasis, are responsible for chemoresistance and poor prognosis in cancer treatments. For instance, pancreatic cancer (Cioffi et al., 2015), hepatic cancer (Govaere et al., 2016) and colorectal cancer (Fesler et al., 2017) were found chemoresistance associated with CSCs.

### 2.7 Refractory to Apoptosis/Cell Death Stimuli

Apoptosis is an intrinsic programmed cell death process contributing to the regulation of important physiological and pathological processes. In apoptosis, activation of caspases, breakdown of DNA and protein and changes of membrane are main biochemical changes (Wong, 2011). Both intrinsic mitochondrial pathway and extrinsic death receptor pathway activate caspases which are both initiators and executioners in the mechanism of apoptosis (Wong, 2011). Apoptosis is recognized as a positive strategy to induce death of cancer cells (Ichim and Tait, 2016). However, malignant cells evade apoptosis to facilitate carcinogenesis and chemoresistance. The inhibition of caspases, suppression of death receptor signaling and disturbed balance of apoptotic proteins including BcI-2 proteins, Mcl-1 proteins, p53 protein, inhibitor of apoptosis proteins (IAPs) are responsible for cancer development and chemoresistance (Wong, 2011; Mohammad et al., 2015; Ichim and Tait, 2016). For instance, cisplatin-resistant ovarian cancer (Yang et al., 2008) and oxaliplatin-resistant colorectal cancer (Choi et al., 2018) was attributed to inhibition of apoptosis-induced death thereby resulting in chemoresistance.

### 2.8 Tumor Dormancy

Tumor dormancy is recently recognized as a tactics of malignant cells to survive, recur and metastasize. In the dormancy state, cancer cells are clinically undetectable, undergoing G1–G0 growth arrest and surviving the exposure to cytotoxic drugs (Yumoto et al., 2014). The dormancy is also a characteristic of self-renew cancer cells like cancer stem sell. Dormancy-competent cancer stem cells are proposed as a neoplastic subpopulation controlling alternative of dormancy and growth (Crea et al., 2015). Remaining in dormancy period suggests these cells are able to regulate their cell cycle. Besides, angiogenic switch, immune escape and cellular dormancy mechanisms contribute to tumor dormancy (Yumoto et al., 2014; Jahanban-Esfahlan et al., 2019). Strikingly, the microenvironment changes play a pivotal role in reactivating dormant cancer cell after chemotherapy to develop chemoresistance (Yumoto et al., 2014; De Angelis et al., 2019). Disturbing their niche to wake up dormant cells then killed by anticancer drugs or to sustain tumor dormancy infinitely is considered to be a strategy to defeat tumor-dormancy-induced chemoresistance (Yumoto et al., 2014; De Angelis et al., 2019).

## 3 Metabolic Reprogramming in Cancer

Metabolic reprogramming is one of emerging hallmarks of cancer (Hanahan and Weinberg, 2011). Tumor cells reside in a microenvironment characterized by hyper-oxidative, hypoxic, acidic and nutrient-poor conditions (Najafi et al., 2019). Metabolic alterations benefit malignant cells to adapt to microenvironment to maintain their survival and growth (Pavlova and Thompson, 2016). More than a century ago, Otto Warburg observed that tumor cells preferred glycolysis to mitochondrial oxidative phosphorylation (OXPHOS) even in oxygen rich conditions, a phenomenon named aerobic glycolysis (Warburg, 1956b; a). This perturbed metabolism imparts tumor cells to make a balance between energy supply and accumulation of glycolytic intermediates for building blocks and NADPH production (Koppenol et al., 2011). Importantly, Warburg effect leads to chemoresistance through epigenetic and genetic changes to activate survival pathway and prevent cell death (Icard et al., 2018). In addition to glucose metabolism alterations, the reprograming of lipid metabolism and perturbation of amino acids metabolisms are also intensively implicated in cancer development and progression (Pavlova and Thompson, 2016). Glutamine, an abundant amino acid in the body and a central precursor for protein in proliferating cells, plays important roles in maintaining intestinal function and amino acid homeostasis (Cluntun et al., 2017). In cancer cells, deregulated uptake of glutamine is a hallmark that cancer cells display a higher consumption of glutamine to satisfy their proliferation (Pavlova and Thompson, 2016). Interestingly, there is an underlying association among glycolysis, rewired amino acids metabolism and altered lipid metabolism which are still being elucidated. For instance, the enzymes in Warburg effect have been reported to be involved in the aberrant lipid metabolism. Pyruvate kinase M2 (PKM2), a major glycolytic enzyme in Warburg effect facilitating chemoresistance (Li et al., 2015; Shanmugasundaram et al., 2017), interacts with sterol regulatory element-binding proteins (SREBPs) to increase nuclear SREBP-1a’s abundance and activate lipogenic gene expression as well as lipid biosynthesis subsequently (Zhao et al., 2018). Besides, altered amino acids metabolism was regulated by transcription factor MYC (Wise et al., 2008), which is also attributed to lipid metabolism reprogramming (Eberlin et al., 2014). Moreover, intermediate substances associate the Warburg effect with the lipid biosynthesis. Glucose metabolism imports citrate to FA metabolism, which is the intersection of the two major metabolic pathways as an intermediate in the Krebs cycle (Currie et al., 2013). A new thesis of mitochondrial OXPHOS and respiration highlighted the significant role of mitochondria in resistant cancer cells (Bosc et al., 2017). The dysfunction of the mitochondria, as a signaling center converging most catabolic and anabolic pathways, contributed to cell survival and chemoresistance.

In terms of lipid metabolism, among the diverse categories of lipid, FA is regarded to be fundamentally involved in the development of malignant biological properties in cancer cells (Currie et al., 2013). In the process of lipid biosynthesis, citrate needs several steps to convert carbons to bioactive FAs. Then, the free FA is esterified to triacylglycerol (TAG) and storage in lipid droplets (LDs). As an integral part of metabolism, lipid metabolism, especially aberrant FA metabolism, is emphasized in tumorigenesis and cancer chemoresistance.

## 4 Fatty Acid Metabolism and Chemoresistance

### 4.1 Lipid Biosynthesis

**FIGURE 2:**
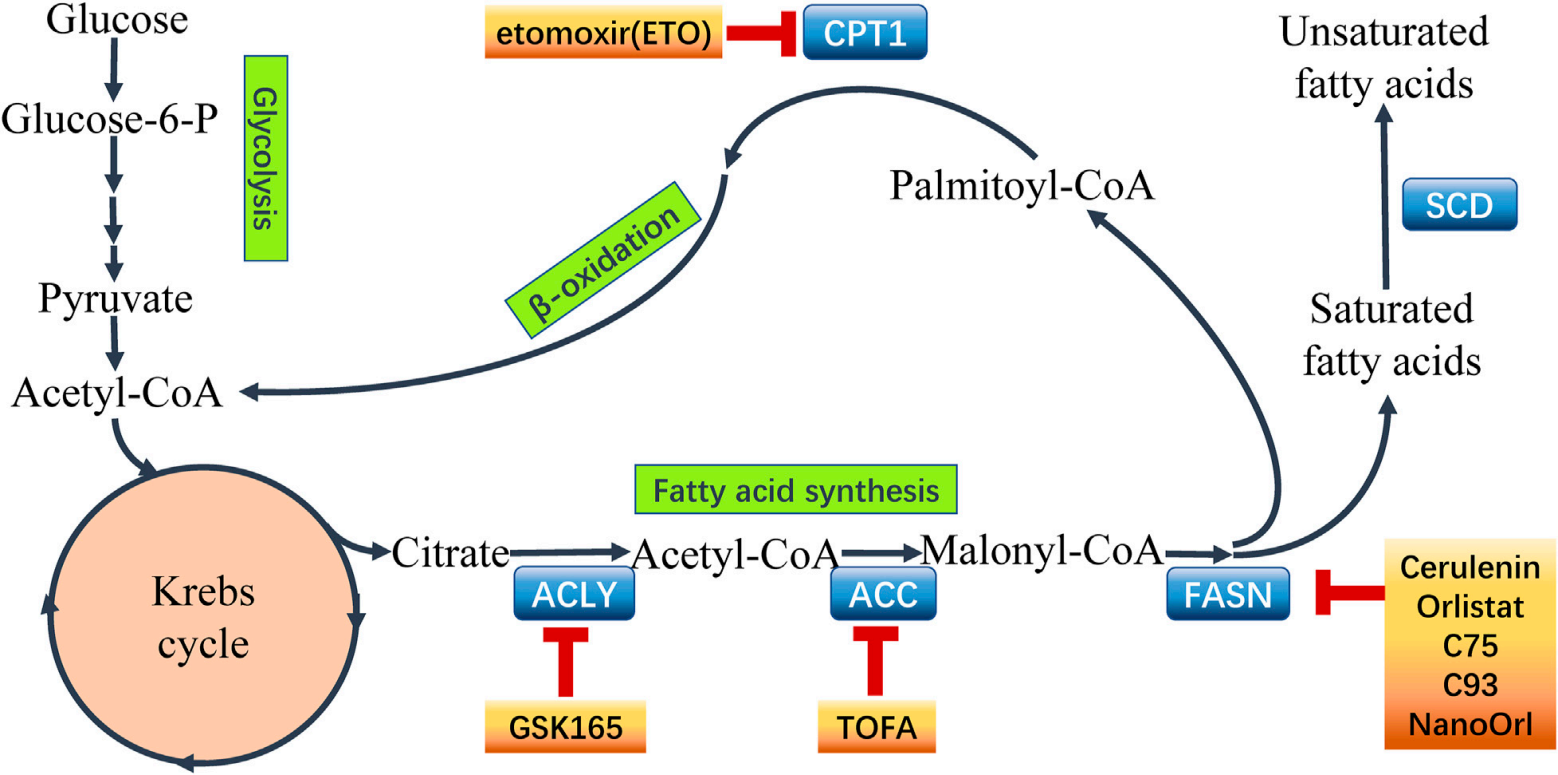
Fatty acid metabolism and points of intervention to overcome chemoresistance. Fatty acid metabolism plays a critical role in chemoresistant cancer cells. Aberrant metabolism contributes to the survival and proliferation of malignant cells. Importantly, the key enzymes catalyzing the processes in fatty acid metabolism have been recognized to express abnormally in cancer chemoresistance. Hence, it is a novel strategy using inhibitors to target the key enzymes to intervene the aberrant metabolism and overcome chemoresistance in cancer. Some common inhibitors of the key enzymes are shown above. ATP-citrate lyase (ACLY), acetyl-CoA carboxylase (ACC), fatty acid synthase (FASN), stearoyl-CoA desaturase (SCD), carnitine palmitoyltransferase 1 (CPT1).

The lipid biosynthesis is an intricate process which is controlled by complex, cross-linked regulatory networks including enormous regulatory factors and regulatory pathways (Grunt, 2018). Enhanced lipogenesis contributes to cancer growth, metastasis and acquisition of drug resistance resulting in unfavorable clinical outcome in cancer patients. Activation of FA synthesis in cancer can be achieved by transcriptional induction. Sterol regulatory element binding proteins (SREBPs) is a master regulators in lipogenetic regulation in cancer (Shao and Espenshade, 2012). SREBPs transcriptionally activates enzymes involved in lipogenesis processes in cancer cells (Liu et al., 2017), including ATP citrate lyase (ACLY), acetyl-CoA carboxylase (ACC), fatty acid synthase (FASN), and acyl-CoA synthetase (ACS) which play significant roles in the process of lipid biosynthesis (Currie et al., 2013) (seen Figure 2). These enzymes are potentially involved in the interaction between the altered lipogenesis and chemoresistance (Roehrig and Schulze, 2016). So far, several attempts are being made to focus on the rewired lipid biosynthesis in order to overcome cancer chemoresistance.

#### 4.1.1 ACLY

ACLY is the rate-limiting enzyme for the first-step of *de novo* lipogenesis. It is a cytosolic homotetrameric enzyme that catalyzes mitochondrial derived citrate into acetyl CoA, the precursor of FA synthesis, thus connecting glucose metabolism to lipid metabolism (Zaidi et al., 2012). ACLY is reported to be up-regulated in many cancer cells such as glioblastoma (Beckner et al., 2010), breast cancer (Lucenay et al., 2016; Wang et al., 2017), colorectal cancer (Zhou et al., 2013), osteosarcoma (Xin et al., 2016), prostate cancer (Xin et al., 2016), cervical cancer (Xin et al., 2016) and lung cancer (Xin et al., 2016), indicating overexpression of ACLY is a hallmark of cancer. Inhibition of ACLY by RNA interference or chemical inhibitor SB-204990 inhibited cancer proliferation (Hatzivassiliou et al., 2005). Activation of Akt pathway may either directly activate ACLY by phosphorylating ACLY protein (Potapova et al., 2000; Berwick et al., 2002), or transcriptionally enhance the expression level of ACLY by SREBP (Porstmann et al., 2005), thus triggering the production of cytosolic acetyl-CoA for lipogenesis and protein acetylation reactions (Hoxhaj and Manning, 2020). The mTORC2/AKt/ACLY pathway (Martinez Calejman et al., 2020) and the PI3K/Akt/ACLY pathway (Lee et al., 2014) are involved in histone acetylation, which plays important roles in gene regulation and DNA damage repair dependent on acetyl-CoA provided by ACLY (Sivanand et al., 2017). The ACLY is highlighted in histone acetylation and carcinogenesis (Carrer et al., 2019), indicating that oncogenic metabolic reprogramming regulated the epigenome (Lee et al., 2014). ACLY is also responsible for increased cancer stemness (Hanai et al., 2013; Zhang et al., 2022), cancer cell adhesion and migration (Lee J. V. et al., 2018; Wen et al., 2019). These characteristics render cancer cells capability in growth as well as chemoresistance.

Zhou *et al.* revealed that upregulation of ACLY confers colorectal cancer cells resistance to SN38, an active ingredient converted from irinotecan (Zhou et al., 2013). ACLY promotes resistance to MAPK inhibitors by increased histone acetylation facilitating MITF-PGC1α axis transcription in melanoma (Guo et al., 2020). In castration-resistant prostate cancer cells, combining androgen receptor (AR) antagonist with ACLY inhibition promoted AMPK activation and lead to suppression of AR levels to induce apoptosis (Shah et al., 2016). Besides, blocking ACLY sensitized cancer cells to chemotherapy by suppressing stem cell features (Hanai et al., 2013). Due to the complicated association between ACLY and signal pathways, combinatory targeting ACLY with other critical molecules may be more effective than using ACLY knockdown alone (Zhou et al., 2013). For example, inhibiting ACLY alone was unefficient to restore chemo-sensitivity to SN38 since compensatory activation of AKT pathway was induced when ACLY was suppressed (Zhou et al., 2013). Combining ACLY inhibition with tyrosine kinase or PI3K/Akt inhibitors could notably abrogate the effects of PI3K/Akt activation in metabolism (Hatzivassiliou et al., 2005). Dual blockade of MAPK and PI3K/AKT pathways by combining ACLY knockdown and statin treatment sensitized EGF receptor resistant in non-small cell lung cancer (NSCLC) (Hanai et al., 2012).

#### 4.1.2 FASN

FASN is the enzyme catalyzing the terminal steps in the *de novo* biosynthesis of long-chain FAs (Menendez and Lupu, 2007). FASN consumes acetyl-CoA, malonyl-CoA and NADPH to catalyze the biosynthesis of palmitate, sustaining altered metabolic state for cancer growth and survival (Jones and Infante, 2015). Palmitate is an important product in *de novo* FA synthesis which possesses vital biological function (Menendez and Lupu, 2007). Palmitate and additional FAs are considered precursors of cellular lipid synthesis for membrane fluidity and function, and substrates of post-translational protein regulation for protein localization and activity (Ventura et al., 2015). Palmitate interacts with lipid rafts, which are specialized plasma membrane microdomains rich in lipid-modified membrane-associated proteins and lipids, to play important roles in receiving, localizing, and transmitting (Simons and Sampaio, 2011). FASN inhibition may affect membrane structure such as lipid rafts, thus disrupting signal transduction networks and biological processes. However, activation of FASN is a hallmark of cancer contributing to tumor growth, invasion and stemness (Yasumoto et al., 2016).

Dysfunction of FASN is reported as a new characteristic in chemoresistance in malignant cells. Studies demonstrated that FASN levels were increased in paclitaxel-resistant hepatocellular carcinoma (HCC) (Meena et al., 2013), taxane-resistant prostate cancer (Souchek et al., 2017), cisplatin-resistant squamous cell carcinoma (Huang et al., 2012), paclitaxel-resistant laryngeal cancer (Xu et al., 2013), gemcitabine-resistant pancreatic cancer (Yang et al., 2011) and carboplatin/paclitaxel-resistant ovarian cancer (Ueda et al., 2010).

In resistant breast cancer cells, increased FASN-catalyzed endogenous FA biogenesis was related to HER2 overexpression (Vazquez-Martin et al., 2008; Puig et al., 2011; Ligorio et al., 2021). In trastuzumab-resistant HER2-positive breast cancer cells, overexpression of FASN was facilitated by Pin1 via regulating EGF signaling pathway to activate the sterol regulatory element-binding protein-1c (SREBP1c) promoter (Yun et al., 2014). Inducing SREBP-1 gene to activate the Akt and HIF1 in breast cancer also increased the expression of FASN, which partly conduced to hypoxia-induced chemoresistance to cyclophosphamide (Furuta et al., 2008). Simultaneous blocking FASN and HER2 pathways showed significantly anti-tumor effect for breast cancer cells resistant to anti-HER2 drugs (Blancafort et al., 2015). In addition, FASN promotes cancer chemoresistance by regulation of apoptosis. FASN overexpression caused palmitate overproduction, thus supporting anti-apoptosis (Liu et al., 2008). Also, FASN overexpression inhibited TNF-α expression, which in turn suppressed NF-κB as well as neutral sphingomyelinase (nSMase) to reduce ceramide production, suppress caspase 8 activation, and inhibit apoptosis (Liu et al., 2013).

In gastric cancer cells, MaCC1, the upstream regulator of FASN, decreased the chemosensitivity to oxaliplatin by enhancing the expression of FASN (Duan et al., 2017). Moreover, in cisplatin-resistant NSCLC, increased FASN expression was regulated by a FASN-TGFβ1-FASN positive feedback loop which mediated epithelial mesenchymal transition (EMT) (Yang et al., 2016). Activation of EGFR/FASN-Akt signaling axis was found in acquired TKI-resistant EGFR mutated NSCLC cells (Ali et al., 2018). In addition to EGFR mutated cancer cells, Akt pathway was related to chemoresistance in FASN-overexpressed gastrointestinal stromal tumors (GISTs) with KIT mutated and the activation of oncogenic pathway like PI3K/AKT/mTOR pathway promoted chemoresistance to imatinib (Li et al., 2017). In gemcitabine-resistant pancreatic cancer cells, a notably increase in FASN expression was closely associated with regulating ER stress, which resulted in apoptosis, and stemness (Tadros et al., 2017). In vemurafenib-resistant BRAF-mutant melanoma, active SREBP-1, the downstream target of BRAF signaling, was the characteristic to sustain lipogenesis and up-regulate the expression of FASN (Talebi et al., 2018).

Targeting FASN by FASN inhibitors is useful to sensitize cancer cells to chemotherapies (seen Figure 2). Since the FASN oncogenicity involves versatile molecules in anti-apoptosis, lipid raft function and central oncogenic pathway activation (Li et al., 2017), the inhibition of FASN can regulate important biological processes. For instance, PIK3/AKT/mTOR pathway, β-catenin signaling and expression of oncogenic effectors like c-Myc were inhibited by FASN inhibitors (Ventura et al., 2015; Li et al., 2017). Proton pump inhibitors (PPIs) effectively suppressed FASN to induce apoptosis and sensitized to doxorubicin in breast cancer (Wang et al., 2021). Meanwhile, through FASN inhibition, the perturbation of lipid raft, an important membrane structure, destroyed the signal transduction networks to affect cell growth and death (Ventura et al., 2015; Li et al., 2017). New FASN inhibitors with less *in-vivo* toxicity like TVB-3166 exhibited potent anti-tumor effects in chemoresistant cancer cells (Ventura et al., 2015).

#### 4.1.3 ACC

ACC, including ACC1 and ACC2, catalyzes acetyl-CoA to form malonyl-CoA, which is the rate-limiting step to synthesize long-chain FA (Ventura et al., 2015). AMPK is a major regulator of ACC in cancer development. AMPK phosphorylated transcriptional regulator such as SREBP1c to inhibit ACC activity and control FA synthesis (Steinberg and Carling, 2019). Importantly, the levels of acetyl-CoA and malonyl-CoA affected the initiation of FA synthesis or fatty acid oxidation (FAO) (Carracedo et al., 2013). FAO leaded to the accumulation of cytoplasmic acetyl-CoA needed for the initiation of the FA synthesis (Caro et al., 2012). The ACC-catalyzed production malonyl-CoA was reported to be an allosteric inhibitor of a key enzyme of FAO--CPT1 (McGarry et al., 1983). Additionally, genetic manipulation of ACC1 or ACC2 disturbed the balance of FA synthesis and FAO in cancer cells (Jeon et al., 2012). It was reported that overexpression of ACC1 in NSCLC plays a crucial role in maintaining cancer cell growth and viability (Svensson et al., 2016). Blocking ACC inhibits tumor growth of NSCLC (Svensson et al., 2016).

Recently, ACC is emphasized in cancer chemoresistance. In cisplatin-resistant lung cancer cells which had high ROS and low thioredoxin-1 (TRX1) levels, ACC presented a significantly high basal level (Wangpaichitr et al., 2012). The up-regulation of the antioxidative factor TRX1 caused an attenuated level of ACC (Wangpaichitr et al., 2012). In cetuximab-resistant head and neck squamous cell carcinoma (HNSCC), an increased level of total ACC regulated by post-translational mechanism compensated the AMPK activation-induced ACC inhibition, thus promoting cancer cells to survive the cetuximab (Luo et al., 2017). Co-targeting ACC with TOFA, an ACC allosteric inhibitor, significantly sensitized cetuximab-resistant HNSCC xenografts to cetuximab (Luo et al., 2017).

### 4.2 Lipid Desaturation

Lipid desaturation is an integral modification in cancer cells to limit lipotoxicity and generate unsaturated FAs (Ackerman and Simon, 2014). Monounsaturated fatty acid (MUFA) is the major product of lipid desaturation to regulate the membrane fluidity, normal cell function and cancer development (Hendrich and Michalak, 2003; Calder, 2015; Igal, 2016). Stearoyl-CoA desaturase (SCD) is the key fatty acyl △9-desaturing enzyme to convert MUFA from saturated fatty acid (SFA). Dysfunction of SCD in cancer yields an altered level of MUFA, which breaks the balance of MUFA/SFA ratio and change the cellular lipid composition, fluidity and signal transduction to promote cell survival and induce cancer chemoresistance. Dietaries involving caloric restriction and a ketogenic diet could impair SCD activity in cancer (Lien et al., 2021).

#### 4.2.1 SCD1

SCD is upregulated in several cancers. Overexpression of SCD enhanced cell viability in breast and prostate cancers (Peck et al., 2016). Elevated level of SCD1 was involved in poor prognosis of bladder cancer (Piao et al., 2018) and clear cell renal cell carcinoma (von Roemeling et al., 2013; Wang et al., 2016). In lung cancer, EGFR-mediated phosphorylation of Y55, a mutant of tyrosine residue, enhanced SCD1 stability and activity, which led to enhanced MUFA synthesis and accelerated cell growth (Zhang et al., 2017). In addition, 17β-estradiol (17β-ED) up-regulated SCD-1 *via* activating SREBP-1c, which increased MUFA/SFA ratio in estrogen-sensitive breast cancer (Belkaid et al., 2015). Inhibition of SREBP1 down-regulates SCD1, which is a potential approach to treat pancreatic cancer (Siqingaowa et al., 2017). Due to the elevated SCD1 activity, cancer cells contain aberrant higher levels of MUFA, which is considered as a hallmark of cancer manifesting a distinctive transformation of lipogenesis (Igal, 2011). The relative abundance of MUFAs of membrane lipids protects cancer cells from ER stress (Griffiths et al., 2013) and apoptosis (Peck et al., 2016).

Overexpression of SCD1 is also responsible for chemoresistance in malignant cells. Studies revealed that up-regulation of SCD1 contributed to liver tumor-initiating cells and sorafenib resistance by regulating ER stress-mediated differentiation (Ma et al., 2017). FBJ murine osteosarcoma viral oncogene homolog B (FOSB) also mediated the SCD-dependent acquire-resistance in glioblastoma cell lines (Oatman et al., 2021). Inhibiting SCD1 activated ER stress response and enhanced autophagy to overcome chemoresistance in cisplatin-resistant lung cancer stem cells (Pisanu et al., 2017). Furthermore, SCD regulated lipid desaturation contributes to maintaining stemness. For example, SCD1 was identified to be an upstream activator of β-catenin and YAP/TAZ pathways (Pisanu et al., 2017). Higher expression of SCD1 increased the activity of YAP/TAZ, a stem cell pathway, which supported the stemness and adapted to BRAF inhibitor (vemurafenib) and MEK inhibitor (binimetinib) treatments in BRAF-mutated melanoma (Pisanu et al., 2018). Moreover, in TMZ-resistant glioma cells, up-regulation of SCD1 accounted for promoting Akt/GSK3β/β-catenin signaling (Dai et al., 2017). JNK and PI3K signaling and SREBP-1 promoted SCD expression to lead chemoresistance in HCC (Bansal et al., 2014). Interestingly, SCD is involved in ferroptosis. In ovarian cancer cells and stem cells, over-expression of SCD1 assisted cancer cells to survive ferroptosis (Tesfay et al., 2019). In this study, coenzyme Q_10_, an endogenous membrane antioxidant involved in ferroptosis, was decreased by SCD1 inhibition thus inducing ferroptosis and apoptosis. Ferroptosis is a novel and unique non-apoptotic cell death form, which is different from apoptosis, necrosis and autophagy, involving iron-dependent ROS formation and lipid oxidation (Yang and Stockwell, 2016). Activation of ferroptosis observed in cancer contributed to destroying the cancer cells (Dixon et al., 2012). However, cancer cells were equipped with potential mechanism to survive the ferroptosis (Lee et al., 2020). During ferroptosis, polyunsaturated fatty acids (PUFAs) in cellular membrane react with ROS to induce lipid peroxidation (Xie et al., 2016). Two lipid metabolism-associated enzymes, lysophosphatidylcholine acyltransferase 3 (ACSL4) and lysophosphatidylcholine acyltransferase 3 (LPCAT3) participate in the formation of membrane phospholipids from PUFAs such as arachidonic acid (AA) or adrenic acid (AdA), thus protecting cancer cells against ferroptosis (Chen et al., 2021; Lee et al., 2021). Exogenous MUFAs activated by ACSL3 could inhibit membrane lipid ROS accumulation and suppress ferroptosis in cancer cells (Magtanong et al., 2019). In the context of chemoresistance, ferroptosis plays an important role. Induction of ferroptosis was reported to overcome drug resistance in head and neck cancer, glioblastoma multiforme and colorectal cancer (Kazan et al., 2017). SCD1 was regulated by complicated pathways associating tumor suppressors such as p53 in ferroptosis (Gnanapradeepan et al., 2018; Angeli et al., 2019). The SCD1 inhibition altered the membrane phospholipid composition, thus influencing ferroptosis (Tesfay et al., 2019). The content of MUFA chains could be displaced by increased PUFA chains, thus facilitating membrane lipid oxidation and ferroptosis (Tesfay et al., 2019; Snaebjornsson et al., 2020).

SCD inhibition suppressed cell proliferation and resensitized cancer cells to chemotherapy-induced apoptosis (Bansal et al., 2014). However, to inhibit lipid desaturation, SCD inhibition is not always efficient. Strikingly, during SCD inhibition, other enzyme such as FA desaturase 2 (FADS2) was utilized by cancer cells to fulfil their requirements for MUFA (Vriens et al., 2019). Plus, the uptake of ample exogenous unsaturated FAs compensated the shortage of lipid desaturation by SCD inhibition (Snaebjornsson et al., 2020).

### 4.3 Lipid Droplets

The lipid droplet (LD), recognized as a multi-functional organelle in most eukaryotic cells, is composed of a neutral lipid core mainly including triacylglycerols (TAGs) and sterol esters (SEs) with a phospholipid monolayer membrane (Farese and Walther, 2009; Walther et al., 2017; Zhang and Liu, 2017). LD fulfills different roles according to cell types and physiological states. It functions as a repository for energy storage and generation, or membrane synthesis (Farese and Walther, 2009; Walther et al., 2017). Accumulation of LDs is emerging as a new hallmark of cancer and cancer stemness.

There exists a close relation between FA metabolism and LDs. The excess FAs generated by lipogenic enzymes are then esterified and stored as TAG and cholesterol esters (CE) in LDs, which help cancer cells evade the harmful effect of the excess free FAs and survive under starvation by liberating FAs from LDs to mobilize into mitochondria for oxidation (Rambold et al., 2015). LD has a complicated relationship with LD-related proteins, enzymes and other organelles such as endoplasmic reticulum (ER), nucleus, mitochondria and peroxisomes (Olzmann and Carvalho, 2019). ER contributes to LD formation by synthesizing and packaging neutral lipids in lens-like structures to bud from its membrane (Barneda and Christian, 2017). The continuity in their membranes supports the growth of LD, which also suggests the direct connection between LD and ER (Barneda and Christian, 2017). This ER-LD contact contributed to the rapid lipid exchange during lipid metabolism between ER and LD (Farese and Walther, 2009). A form of this contact was a membrane bridge which supports connection and allowed TG synthesis enzymes migrate to LD surface (Walther et al., 2017), which may participate in the local synthesis of TG (Farese and Walther, 2009). In addition to the connection with ER, LD interacted closely with mitochondria to transfer FAs for β-oxidation (Rambold et al., 2015).

It has been recently demonstrated that aberrant accumulation of LDs appeared in *KRAS* and *BRAF* mutated colon cancer cells, which had poor response to erlotinib, an EGFR-inhibition treatment (Yosef et al., 2015). Besides, progestin facilitated accumulation of LDs in PR^+^ breast cancers, which in turn caused docetaxel sequestered in enlarged LDs to prevent docetaxel binding to microtubule (Schlaepfer et al., 2012). Similarly, sequestration of prodrug CHR2863 (a hydrophobic aminopeptidase inhibitor) in accumulated LDs suppressed the conversion from CHR2863 to its acid metabolite CHR6768 to promote chemoresistance in myeloid leukemia cells (Verbrugge et al., 2016). Besides, ponatinib, a multi-tyrosine kinase inhibitor, was deposited into increased LDs to influence intracellular pharmacokinetics and then induce chemoresistance in lung cancer (Englinger et al., 2020). Collectively, it has been suggested that drugs with dominant hydrophobicity may be sequestrated into the accumulative LDs to block the cell-killing effects of chemotherapies, which is utilized by cancer cells to accumulate LDs sequestering anti-cancer agents to avoid death. This may be a mechanism for cancer cells to reduce cytoplasmic drug concentrations, and targeting LD integrity will be a novel strategy to deal with the chemoresistance of lipophilic anticancer drugs.

In addition to the hydrophobic characteristic of drugs, the participation of other enzymes, molecules and organelles contributes to LD-associated chemoresistance. In ovarian and cervical cancer, a higher level of LDs was found in resistant cell lines, which was associated with the increased activity of 6-phosphofructo-2-kinase/fructose-2,6-biphosphatase 3 enzyme (PFKFB3), a glycolytic regulator that also regulated a key step to control glycolytic rate by mediating fructose-2,6-bisphosphate (F2,6BP) levels (Mondal et al., 2019). PFK158, a PFKFB3 inhibitor sensitized cancer cells by inducing LDs inhibition, which was related to an up-regulation of autophagic flux to boost lipophagy and induce apoptosis for suppression of tumorigenesis (Mondal et al., 2019). Moreover, an LD-localized enzyme called lysophosphatidylcholine acyltransferase 2 (LPCAT2) was reported to contribute to LD-induced chemoresistance of 5-fluorouracil and oxaliplatin by inhibiting caspase cascade activation as well as ER stress responses mechanistically (Cotte et al., 2018).

Since LDs contain different neutral lipids, it is important to clarify the precise effect of these components and networks. Not only FA metabolism but also cholesterol metabolism is emphasized in LD-induced chemoresistance. It has been shown that enhanced LDs and cholesterol esters were observed in carboplatin-resistant laryngeal carcinoma cells (Rak et al., 2014). Moreover, both increased cholesterol esters and triglycerides accumulating in LDs promoted breast cancer cells to survive the tamoxifen treatment (Hultsch et al., 2018). In this study, downregulated lysosomal protein cathepsin D and enhanced lysosomal-associated-membrane-proteins 1 and 2 suppressed lysosomal membrane permeabilization to prevent tamoxifen-resistant breast cancer cells from lysosomal cell death.

### 4.4 Lipid Catabolism

Lipid catabolism, also known as β-oxidation, yields ATP, NADH and acetyl-CoA to maintain energy balance, reduce oxidative stress and support (histone) protein acetylation (Grunt, 2018). Enhanced FAO not only increases ATP production but also provides NADH and FADH2 thus rendering metabolic advantages to tumor cells (Carracedo et al., 2013). FAO product Acetyl-CoA enters various metabolic pathways and contributes to FA synthesis, TCA cycle, and protein acetylation (Yoshii et al., 2015). The carnitine palmitoyltransferases (CPTs), two forms of mitochondrial membrane-bound enzymes named CPT1 and CPT2, function to uptake FAs across the mitochondrial membrane and play critical role in the regulation of FAO in cells (seen Figure 2). Recent studies have recognized that FAO occupies a critical position in rewiring FA metabolism to contribute to therapy resistance in cancer cells.

#### 4.4.1 CPT

CPT1 is the key enzyme located on outer mitochondrial membrane to regulate the entry of FAs into the mitochondria via converting FA-CoAs to FA carnitines (Currie et al., 2013). CPT1a, CPT1b and CPT1c are three subtypes of CPT family showing specific tissue distribution (Qu et al., 2016). CPT1 antagonized apoptosis and supported cancer cell to survive the metabolic stress (Qu et al., 2016). CPT1a contributed to metastasis of colorectal cancer cell by anoikis inhibition (Wang Y.-n. et al., 2018). CPT1b was upregulated by phosphatidylinositol transfer protein cytoplasmic 1 (PITPNC1) to promote FAO and anoikis resistance thus facilitating omental metastasis of gastric cancer (Tan et al., 2018). In addition, CPT1 is responsible for cancer chemoresistance. The high levels of CPT-1a in pancreatic ductal adenocarcinoma (PDAC) patients was associated with chemoresistance by rewiring cancer lipid metabolism to escape from energy stress (Luo et al., 2016). Activation of FAO provides cancer cells sufficient energy to survive the metabolic stress induced by chemotherapies. The mammary-adipocyte-derived leptin activated STAT3 signaling to promote stemness and chemoresistance through transcriptionally induction of CPT-1b and activating FAO in breast cancer cells (Wang T. et al., 2018). Silencing STAT3 impaired FAO and inhibited self-renewal, whereas stimulating FAO by bezafibrate rescued self-renewal in breast cancer stem cells (Wang T. et al., 2018). Blocking FAO by CPT1 inhibitor etomoxir re-sensitized breast cancer cells to chemotherapy, and inhibited BCSCs in mouse breast tumors *in vivo* (Wang T. et al., 2018). Furthermore, using etomoxir suppressed hypoxia-activated-FAO pathway in anti-angiogenic drug (AAD) treated tumors, and it was considered that the loss of CPT1a function enhanced AAD sensitivity in steatotic liver cancer (Iwamoto et al., 2018) (seen Figure 2).

Compared with CPT1, CPT2 is little known. CPT2 converts FA carnitines back to FA-CoAs located inside the matrix (Lodhi and Semenkovich, 2014). As the other genetically distinct mitochondrial membrane-bound enzyme regulating FAO, CPT2 appears to play a controversial role in chemoresistance. Lower expression of CPT2 is associated with tumor histological differentiation and venous invasion (Lin et al., 2018). Silencing CPT2 not only enhances the tumorigenic activity and metastatic potential, but also induces chemoresistance to cisplatin in hepatoma cells (Lin et al., 2018). Decrease of CPT2 leads to upregulation of SCD1 and enhanced lipogenesis in HCC cells, indicating that CPT2 may be a key regulator in SCD1-mediated lipid metabolism in drug resistant cancer cells (Lin et al., 2018). To explain the contradiction that CPT1 and CPT2, which are both key enzymes to promote FAO, play opposite roles in cancer chemoresistance, it is speculated that in some certain conditions CPTs are suppressed to attenuate lipid degradation and maintain sufficient lipid for cancer progression (Lin et al., 2018), although the mechanism is unclear. Conversely, in oxaliplatin-treated gastrointestinal cancer cells, higher expression of CPT2 resulted in poor chemotherapy outcomes (Wang et al., 2020). Mechanistically, nuclear factor of activated T cells 3 (NFATc3) promoted CPT2 expression transcriptionally, and inhibiting CPT2 resensitized cancer cells to oxaliplatin (Wang et al., 2020).

Importantly, Chia-Lin Chen *et al.* demonstrated that NANOG, reprogramming of mitochondrial metabolism, contributed to human tumor-initiating stem-like cells (TICs) oncogenicity and chemoresistance (Chen et al., 2016). NANOG, which mediated oncogenic pathways including stimulating fatty acid oxidation (FAO) and suppressed oxidative phosphorylation (OXPHOS) through mitochondrial metabolic reprogramming, inhibited chemotherapy-induced apoptosis by suppressing ROS production to promote chemoresistance of tumor-initiating stem-like cells (TICs) (Chen et al., 2016). Moreover, in CD36^+^ chemoresistant leukemic stem cells (LSCs), a higher FAO rate was found, indicating a close relationship between FAO and chemoresistance (Ye et al., 2016). Besides, Thomas Farge *et al.* illustrated that CD36–FAO–OXPHOS axis in AML was responsible for cytarabine (AraC) resistance, with an upregulation of CD36 and FAO to maintain high OXPHOS state to survive the chemotherapy (Farge et al., 2017).

In addition to CPT1 and CPT2, master factors involved in lipid metabolism, including peroxisome proliferator-activated receptors (PPARs) and peroxisome proliferator-activated receptor gamma co-activator-1 (PGC-1), are associated with chemoresistance via modulating FAO. There are three isoforms of PPARs, including alpha, beta/delta and gamma belonging to the nuclear receptor superfamily which are modulated differentially by endogenous ligands, drugs and other xenobiotics (Peters et al., 2012). In terms of the role of PPARs in cancer development, it remains controversial that PPARs promote or inhibit cancer growth under certain conditions with complex underlying mechanisms [reviewed in Peters et al. (2012), Peters et al. (2015)]. PPARα was found to regulate the transcription of CPT II gene (Barrero et al., 2003), which confirmed that PPARs have an important effect on controlling FAO enzymes expression. In glucocorticoid (GC)-resistant chronic lymphocytic leukemia (CLL), GCs enhanced PPARα expression and stimulated PPARα-mediated FAO, promoting cell survival upon metabolic stress (Tung et al., 2013). Besides PPARs, the nuclear cofactors regulating mitochondrial oxidative metabolism including transcriptional co-activators PGC-1 family also contribute to cancer chemoresistance. For instance, PML-PGC-1α axis was activated by oxidative stress in high-grade serous ovarian cancer (HGSOC) with high-OXPHOS features, which promoted the sensitivity to conventional chemotherapies (Gentric et al., 2018). PGC-1α promoted metformin-resistance in lung metastatic breast cancer and stimulated bioenergetic flexibility (Andrzejewski et al., 2017).

## 5 Strategies Targeting FA Metabolism to Overcome Chemoresistance

**FIGURE 3:**
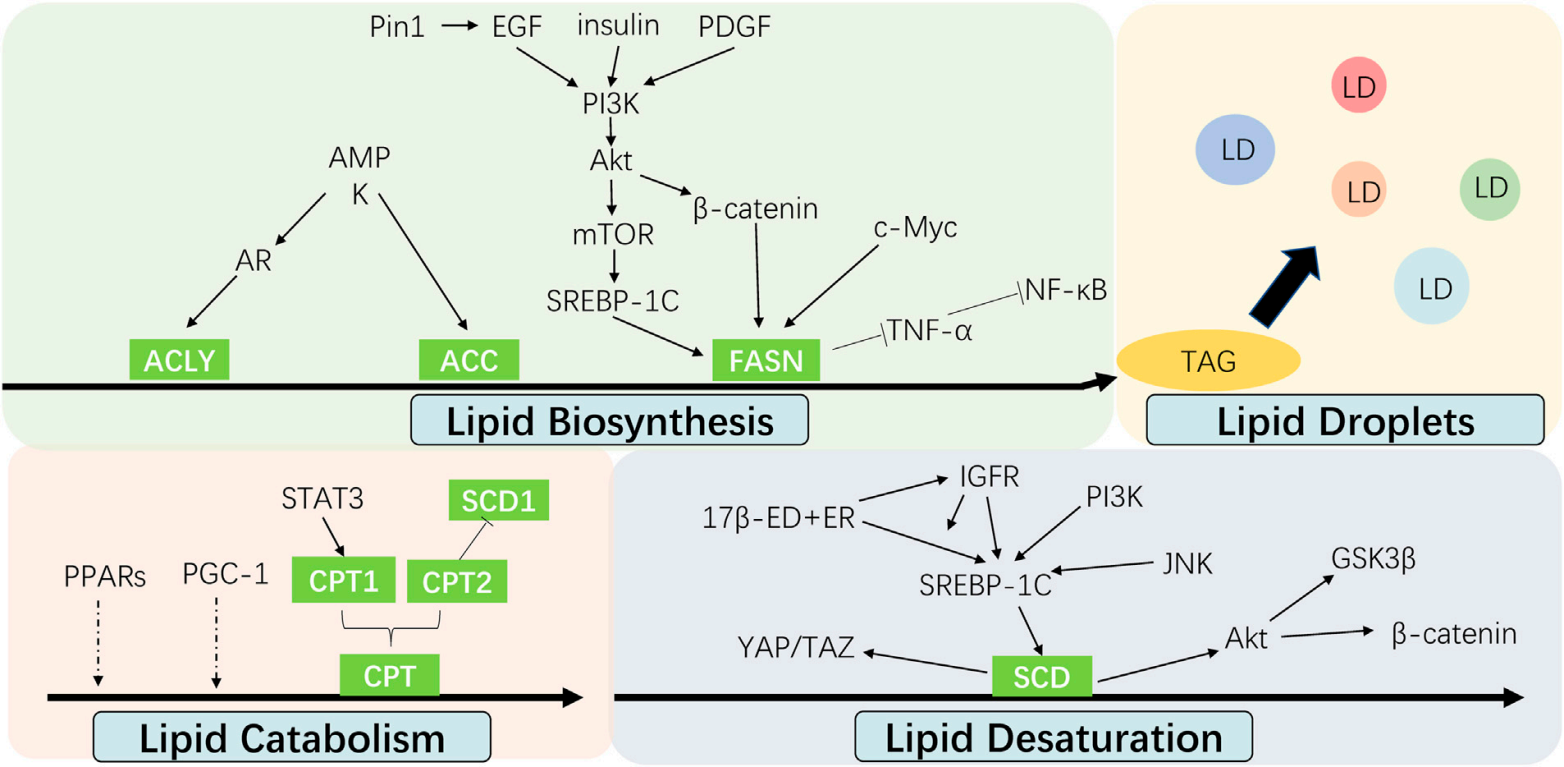
Interaction between cell signalings and altered fatty acid metabolism in cancer chemoresistance. The alteration of fatty acid metabolism including lipid biosynthesis, lipid desaturation, lipid droplets formation and lipid catabolism contributes to cancer chemoresistance, which are regulated by multiple signal molecules.

Recent investigation into the altered FA metabolism in chemoresistant cancer cells (seen Figure 3) has revealed a new insight to address cancer therapy by targeting these key enzymes and pathways in different processes of FA metabolism (seen Figure 2). A summary of the small molecules targeting the key enzymes in FA metabolism are shown in Supplementary Table S1.

Apart from the chemotherapy, immunotherapy is a breakthrough for cancer treatment, which activates natural defenses to eliminate malignant cells (Zhang and Zhang, 2020). PD-1/PD-L1 antibody and chimeric antigen receptor T cells therapy (CART) are immunotherapies developed and approved for clinical use (O'Donnell et al., 2019; Riley et al., 2019; Kennedy and Salama, 2020). It also shed new light into conventional chemotherapeutics. Cioccoloni et al. (2020) found that the overexpressed FASN was associated with the increased PD-L1 expression in a human T-cell leukemia line, and blocking the FASN by Orlistat impaired the expression of the PD-L1. PD-L1 with highly palmitoylation was observed in cisplatin-resistant BC bladder cancer cells (Shahid et al., 2020). The expression and palmitoylation of FASN could increase PD-1 expression and palmitoylation (Shahid et al., 2020). These close associations between lipid metabolism and PD-L1 expression have opened up the opportunity to use the combination of chemotherapies and immunotherapies to overcome cancer chemoresistance.

To improve the bioavailability and tolerance of drugs, recent advances in nanotechnology promote the development of nanoparticle-based drug delivery systems, which is widely used in cancer chemotherapy (Lepeltier et al., 2020; Su et al., 2021). Nanoparticles have advantages including good biocompatibility, low toxicity, long circulation time, controlled release, tumor targeting ability, etc., which is a promising strategy to treat chemoresistant cancer precisely. Additionally, exosomes, the natural subcellular vesicles, is recognized as novel drug carriers. The modified exosomes that wrapping small-molecule chemical drugs can reach the target cells to achieve personalized medicine (Liu et al., 2021). Dan Lin et al. found that iRGD-modified exosomes reversed oxaliplatin resistance of colon by the successful targeted delivery of CPT1A siRNA (Lin et al., 2021).

## 6 Conclusion and Perspective

Lipid metabolism alteration in cancer has provided opportunities for cancer treatment. This review focus on the current knowledge of altered lipid metabolism especially the fatty acids metabolism. We have exhibited various experimental findings and theoretical arguments to show that the alteration of lipid metabolism has crucial effect on chemoresistance in cancer cells. However, many elusive challenges still exist due to the unclear mechanism and the pitfalls of these inhibitors. First, different cancer types may present different sensitivity to the inhibitors, which need biomarkers to predict sensitivity in chemoresistant malignant cells to guide the treatment. The high plasticity of the metabolic network in chemoresistant cancer also bother the treatment by inducing compensatory routes of biosynthesis to maintain survival status, which emphasizes the advantage of combination of multiple treatment targets or approaches. In addition, although some molecules have entered clinical trials such as FASN inhibitor Omeprazole (Sardesai et al., 2021), most of the studies were performed *in vitro* with several limitations and more data *in vivo* is necessary. Drug efficacy and safety need to be further tested by clinical trials.
