# Supplementary material for: Novel Insights on Lipid Metabolism Alterations in Drug Resistance in Cancer
Source: Front Cell Dev Biol. 2022 May 13;10:875318. doi: 10.3389/fcell.2022.875318 (PMC9136290; doi:10.3389/fcell.2022.875318)
Supplement: Supplementary file 1 [file DataSheet1.docx]

Supplementary Material

**Table S1. Inhibitors for fatty acid metabolism to overcome chemoresistance.**

| **Enzyme Target** | **Inhibitor** | **Cancer Model** | **Effect** | **Selected Reference** |
| --- | --- | --- | --- | --- |
| FASN | Cerulenin | cyclophosphamide- resistant breast cancer cells | suppressing the expression of FASN and inducing apoptosis | ([Furuta et al., 2008](#_ENREF_4)) |
|  |  | cisplatin-resistant ovarian cancer cells | suppressing the expression of FASN and inducing apoptosis | ([Bauerschlag et al., 2015](#_ENREF_2)) |
|  |  | paclitaxel-resistant hepatocellular carcinoma cells |  | ([Meena et al., 2013](#_ENREF_10)) |
|  | C75 | oxaliplatin-resistant gastric cancer cells | suppressing the expression of FASN to induce apoptosis or DNA-damage | ([Duan et al., 2017](#_ENREF_3)) |
|  |  | imatinib-resistant gastrointestinal stromal tumors (GIST) with mutant KIT | suppressing *KIT* transactivation and inhibiting the anti-apoptotic PI3K/AKT/mTOR pathway | ([Li et al., 2017](#_ENREF_6)) |
|  | C93 | carboplatin/paclitaxel-resistant ovarian cancer cells | inducing massive apoptosis | ([Ueda et al., 2010](#_ENREF_15)) |
|  | Orlistat | gemcitabine-resistant pancreatic cancer cells |  | ([Yang et al., 2011](#_ENREF_17)) |
|  |  | adriamycin-resistant breast cancer cells | suppressing the expression of FASN and inducing apoptosis | ([Liu et al., 2008](#_ENREF_7)) |
|  |  | cisplatin-resistant T cell lymphoma cells to | modulating cell survival, tumor microenvironment and MDR phenotype. | ([Kant et al., 2014](#_ENREF_5)) |
|  |  | gemcitabine-resistant pancreatic cancer cells | inducing apoptosis  via the ER stress to diminish stemness | ([Tadros et al., 2017](#_ENREF_14)) |
|  |  | TKI-resistant EGFR mutant NSCLC | triggering apoptosis by affecting EGFR signaling | ([Ali et al., 2018](#_ENREF_1)) |
|  |  | cisplatin-resistant ovarian cancer cells | increasing tumor growth delay and inducing apoptosis and necrosis | ([Papaevangelou et al., 2018](#_ENREF_11)) |
|  | NanoOrl (nanoparticles of orlistat) | taxane-resistant (TxR) prostate cancer cells | inducing apoptosis synergistically with docetaxel | ([Souchek et al., 2017](#_ENREF_13)) |
|  | TVB-3166 |  | Suppressing cell growth and inducing apoptosis by perturbation of lipid raft and inhibition of signaling pathways including PI3K–AKT–mTOR and β-catenin | ([Ventura et al., 2015](#_ENREF_16)) |
| ACLY | GSK165 | resistant colorectal cancer cells to SN38 | suppressing the expression of ACLY to activate AKT | ([Zhou et al., 2013](#_ENREF_18)) |
| ACC | 5-(tetradecyloxy)-2-furoic acid (TOFA) | cetuximab-resistant head and neck squamous cell carcinoma (HNSCC) |  | ([Luo et al., 2017](#_ENREF_8)) |
| SCD | MF-438 | Vemurafenib- and binimetinib-resistant BRAF-mutated melanoma | Activating YAP/TAZ to support stemness and promote chemoresistance | ([Pisanu et al., 2018](#_ENREF_12)) |
| CTP-1 | etomoxir (ETO) | gemcitabine-resistant pancreatic ductal adenocarcinoma | inducing apoptosis to lead cells to death | ([Luo et al., 2016](#_ENREF_9)) |

# References:

Ali, A., Levantini, E., Teo, J.T., Goggi, J., Clohessy, J.G., Wu, C.S., et al. (2018). Fatty acid synthase mediates EGFR palmitoylation in EGFR mutated non-small cell lung cancer. doi: 10.15252/emmm.201708313.

Bauerschlag, D.O., Maass, N., Leonhardt, P., Verburg, F.A., Pecks, U., Zeppernick, F., et al. (2015). Fatty acid synthase overexpression: target for therapy and reversal of chemoresistance in ovarian cancer. *J Transl Med* 13**,** 146. doi: 10.1186/s12967-015-0511-3.

Duan, J., Chen, L., Zhou, M., Zhang, J., Sun, L., Huang, N., et al. (2017). MACC1 decreases the chemosensitivity of gastric cancer cells to oxaliplatin by regulating FASN expression. *Oncol Rep* 37(5)**,** 2583-2592. doi: 10.3892/or.2017.5519.

Furuta, E., Pai, S.K., Zhan, R., Bandyopadhyay, S., Watabe, M., Mo, Y.Y., et al. (2008). Fatty acid synthase gene is up-regulated by hypoxia via activation of Akt and sterol regulatory element binding protein-1. *Cancer Res* 68(4)**,** 1003-1011. doi: 10.1158/0008-5472.can-07-2489.

Kant, S., Kumar, A., and Singh, S.M. (2014). Tumor growth retardation and chemosensitizing action of fatty acid synthase inhibitor orlistat on T cell lymphoma: implication of reconstituted tumor microenvironment and multidrug resistance phenotype. *Biochim Biophys Acta* 1840(1)**,** 294-302. doi: 10.1016/j.bbagen.2013.09.020.

Li, C.F., Fang, F.M., Chen, Y.Y., Liu, T.T., Chan, T.C., Yu, S.C., et al. (2017). Overexpressed Fatty Acid Synthase in Gastrointestinal Stromal Tumors: Targeting a Progression-Associated Metabolic Driver Enhances the Antitumor Effect of Imatinib. *Clin Cancer Res* 23(16)**,** 4908-4918. doi: 10.1158/1078-0432.ccr-16-2770.

Liu, H., Liu, Y., and Zhang, J.T. (2008). A new mechanism of drug resistance in breast cancer cells: fatty acid synthase overexpression-mediated palmitate overproduction. *Mol Cancer Ther* 7(2)**,** 263-270. doi: 10.1158/1535-7163.mct-07-0445.

Luo, J., Hong, Y., Lu, Y., Qiu, S., Chaganty, B.K., Zhang, L., et al. (2017). Acetyl-CoA carboxylase rewires cancer metabolism to allow cancer cells to survive inhibition of the Warburg effect by cetuximab. *Cancer Lett* 384**,** 39-49. doi: 10.1016/j.canlet.2016.09.020.

Luo, J., Hong, Y., Tao, X., Wei, X., Zhang, L., and Li, Q. (2016). An indispensable role of CPT-1a to survive cancer cells during energy stress through rewiring cancer metabolism. *Tumour Biol*. doi: 10.1007/s13277-016-5382-6.

Meena, A.S., Sharma, A., Kumari, R., Mohammad, N., Singh, S.V., and Bhat, M.K. (2013). Inherent and acquired resistance to paclitaxel in hepatocellular carcinoma: molecular events involved. *PLoS One* 8(4)**,** e61524. doi: 10.1371/journal.pone.0061524.

Papaevangelou, E., Almeida, G.S., Box, C., deSouza, N.M., and Chung, Y.L. (2018). The effect of FASN inhibition on the growth and metabolism of a cisplatin-resistant ovarian carcinoma model. *Int J Cancer* 143(4)**,** 992-1002. doi: 10.1002/ijc.31392.

Pisanu, M.E., Maugeri-Sacca, M., Fattore, L., Bruschini, S., De Vitis, C., Tabbi, E., et al. (2018). Inhibition of Stearoyl-CoA desaturase 1 reverts BRAF and MEK inhibition-induced selection of cancer stem cells in BRAF-mutated melanoma. *J Exp Clin Cancer Res* 37(1)**,** 318. doi: 10.1186/s13046-018-0989-7.

Souchek, J.J., Davis, A.L., Hill, T.K., Holmes, M.B., Qi, B., Singh, P.K., et al. (2017). Combination Treatment with Orlistat-Containing Nanoparticles and Taxanes Is Synergistic and Enhances Microtubule Stability in Taxane-Resistant Prostate Cancer Cells. *Mol Cancer Ther* 16(9)**,** 1819-1830. doi: 10.1158/1535-7163.mct-17-0013.

Tadros, S., Shukla, S.K., King, R.J., Gunda, V., Vernucci, E., Abrego, J., et al. (2017). De Novo Lipid Synthesis Facilitates Gemcitabine Resistance through Endoplasmic Reticulum Stress in Pancreatic Cancer. *Cancer Res* 77(20)**,** 5503-5517. doi: 10.1158/0008-5472.can-16-3062.

Ueda, S.M., Yap, K.L., Davidson, B., Tian, Y., Murthy, V., Wang, T.L., et al. (2010). Expression of Fatty Acid Synthase Depends on NAC1 and Is Associated with Recurrent Ovarian Serous Carcinomas. *J Oncol* 2010**,** 285191. doi: 10.1155/2010/285191.

Ventura, R., Mordec, K., Waszczuk, J., Wang, Z., Lai, J., Fridlib, M., et al. (2015). Inhibition of de novo Palmitate Synthesis by Fatty Acid Synthase Induces Apoptosis in Tumor Cells by Remodeling Cell Membranes, Inhibiting Signaling Pathways, and Reprogramming Gene Expression. *Ebiomedicine* 2(8)**,** 808-824. doi: 10.1016/j.ebiom.2015.06.020.

Yang, Y., Liu, H., Li, Z., Zhao, Z., Yip-Schneider, M., Fan, Q., et al. (2011). Role of fatty acid synthase in gemcitabine and radiation resistance of pancreatic cancers. *Int J Biochem Mol Biol* 2(1)**,** 89-98.

Zhou, Y., Bollu, L.R., Tozzi, F., Ye, X., Bhattacharya, R., Gao, G., et al. (2013). ATP citrate lyase mediates resistance of colorectal cancer cells to SN38. *Mol Cancer Ther* 12(12)**,** 2782-2791. doi: 10.1158/1535-7163.mct-13-0098.
